# Oxidative Stress and Inflammation Interaction in Ischemia Reperfusion Injury: Role of Programmed Cell Death

**DOI:** 10.1155/2019/6780816

**Published:** 2019-04-07

**Authors:** Weifeng Yao, Lydia Wai Tai, Yiwei Liu, Ziqing Hei, Haobo Li

**Affiliations:** ^1^Department of Anesthesiology, The Third Affiliated Hospital of Sun Yat-sen University, Guangdong 510630, China; ^2^University of Hong Kong, Pokfulam, Hong Kong; ^3^St. Jude Children's Research Hospital, Memphis 38105, USA; ^4^Corrigan-Minehan Heart Center and Cardiology Division, Massachusetts General Hospital, Harvard Medical School, Boston, MA 02114, USA

Ischemia-reperfusion-induced tissue injuries and organ failure are key contributors to postoperative mortality and morbidity [1]. Different types of programmed cell death mediated by oxidative stress and inflammation play critical roles in this pathology [2].

Initiation of programmed cell death by reactive oxygen species- (ROS-) induced oxidative stress plays important roles in ischemia-reperfusion injury (IRI) in multiple organs [3]. In this special issue, W. Yao et al. reported that oxidative stress induced hepatic cell apoptosis and liver dysfunction after orthotopic autologous liver transplantation (OALT), while inhibiting oxidative stress with either anesthetic propofol or selective NADPH oxidase inhibitor VAS2870, reduced hepatic cell apoptosis, and attenuated liver injury after OALT. Further, the induction of pyroptosis and disruption of autophagy also play critical roles in organ dysfunction as reported in this special issue. G.-H. Zheng et al. demonstrated that the induction of pyroptotic cell death mediated via nod-like receptor protein-3 (NLRP3) inflammasome is critical in high-fat diet-induced kidney damage and that the downregulation of vascular endothelial growth factor receptor-2 (VEGFR2) not only reduced NLRP3 activation but also attenuated kidney injury in high-fat diet-treated mice. Moreover, Y. Wang et al. reported that in mouse transient global cerebral ischemia (TGCI), decreased autophagy was accompanied by increased apoptosis in neurons that were associated with neurological dysfunction. Lentiviral or specific agonist overexpression of transient receptor potential mucolipin-1 (TRPML1) enhanced autophagy but inhibited apoptosis and attenuated neurological dysfunction in TGCI.

The importance of oxidative stress in the cause of IRI has been well recognized [4]. Thus, therapies that reduce oxidative stress either by increasing antioxidant capacity or by decreasing ROS production may protect organs against IRI. In this special issue, W. Yao et al. reported that pretreatment with intravenous anesthetic propofol, a potent ROS scavenger that protects the heart against IRI, mitigated OALT-induced cell apoptosis and liver injury. Moreover, G.-H. Zheng et al. reported that purple sweet potato color, a natural anthocyanin, conferred its renal protection through reducing ROS and the subsequent NLRP3 activation in high-fat diet-treated mice. On the other hand, Y. Wang et al. showed that microRNAs (miR-214 and miR-133a), delivered by bone marrow mesenchymal stem cell-derived exosomes, by downregulating CaMKII (by miR-214) and phospholamban (by miR-133a), reduced cardiac stem cell apoptosis under oxidative stress.

Aggravation and persistence of inflammation, secondary to oxidative stress, have been shown to induce programmed cell death and contribute to IRI and organ injury [5]. In this special issue, G.-H. Zheng et al. reported that 20 weeks of high-fat diet treatment-induced ROS overproduction and inflammation, which subsequently activates NLRP3 (mediator of inflammation-induced pyroptosis), leads to kidney injury. Treatment with *α*-lipoic acid, a ROS scavenger, not only mitigated high-fat diet-induced inflammation but also deactivated NLRP3 and attenuated kidney damage. Similarly, W. Yao et al. reported in this special issue that OALT-induced oxidative stress and inflammation were associated with hepatic apoptosis and liver damage and that the application of propofol or NADPH oxidase inhibitor reduced oxidative stress and inflammation, attenuating liver damage. These findings suggest the potential interaction of oxidative stress and inflammation in the induction of programmed cell death during IRI. It is worth noticing that attempts to attenuate organ injury by solely decreasing inflammation or increasing antioxidant capacity have achieved limited success [6, 7], indicating that multifaceted therapies combining anti-inflammatory and antioxidant approaches may be necessary for effective treatment. Thus, further in-depth studies regarding the interaction of inflammation and oxidative stress in the setting of IRI may facilitate our understanding of the pathophysiology of IRI and the development of new therapies for the disease.

We hope that the research articles presented in this special issue contribute to the understanding of current advancements and the mechanisms of oxidative stress and inflammation-mediated programmed cell death in IRI. It is also our hope to stimulate further efforts in the pathological investigation of IRI and the development of therapy for this disease.

We would like to express our gratitude toward the authors for submitting their exciting research for consideration for publication and to the reviewers for sharing their expertise and precious time in reviewing the manuscripts. Thanks to all of those who contributed to the success of this special issue.
